# Supplementary material for: Production of Multifunctional Hydrolysates from the Lupinus mutabilis Protein Using a Micrococcus sp. PC7 Protease
Source: BioTech (Basel). 2025 Apr 27;14(2):32. doi: 10.3390/biotech14020032 (PMC12101353; doi:10.3390/biotech14020032)
Supplement: Supplementary file 1 [file biotech-14-00032-s001.zip › biotech-3523244-supplementary/biotech-3523244-supplementary.pdf]

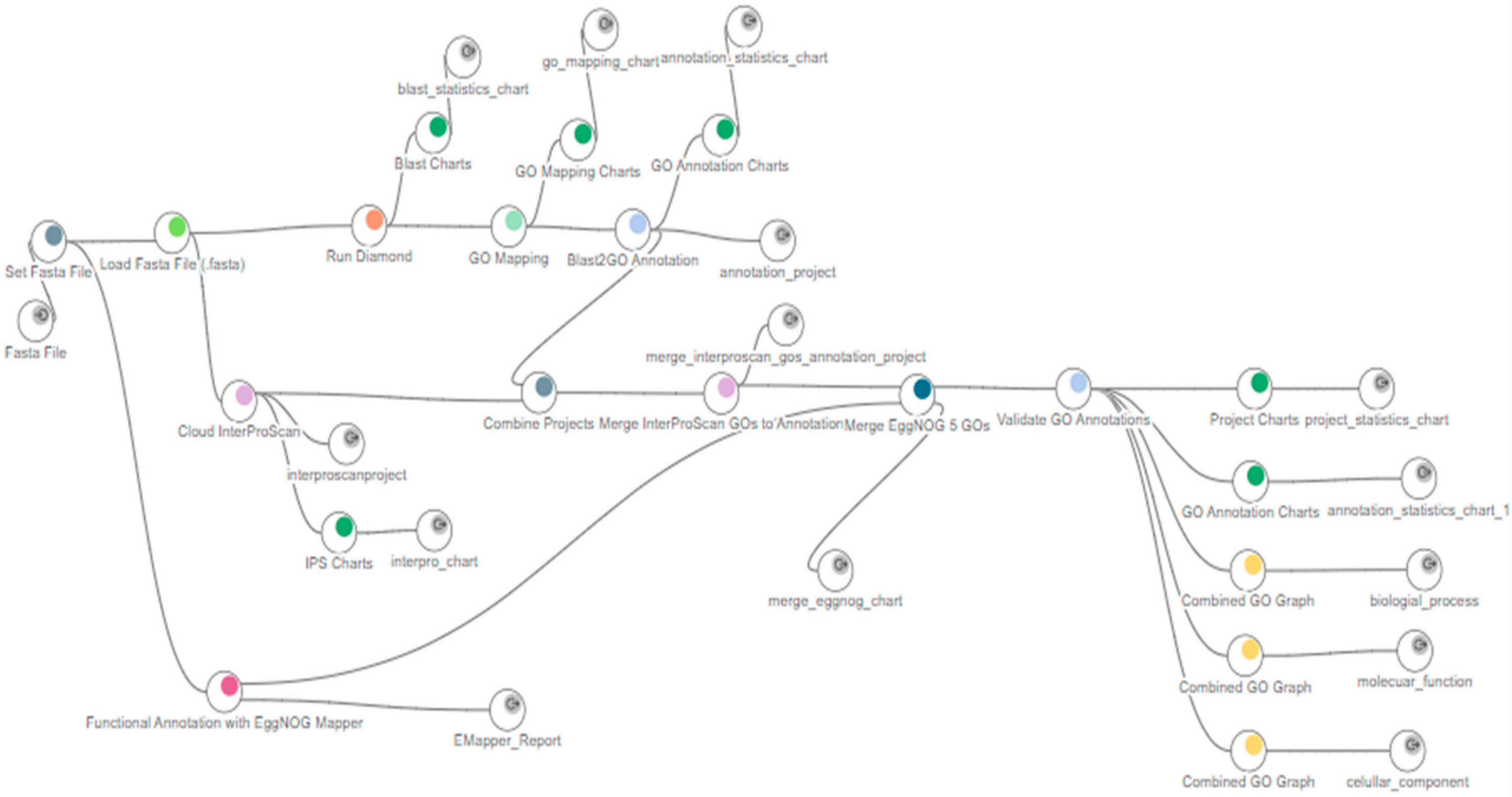

Figure S1: Complete Omicbox workflow followed for the proteomic functional analysis of the albumin fraction of *Lupinus mutabilis* “Tarwi”.

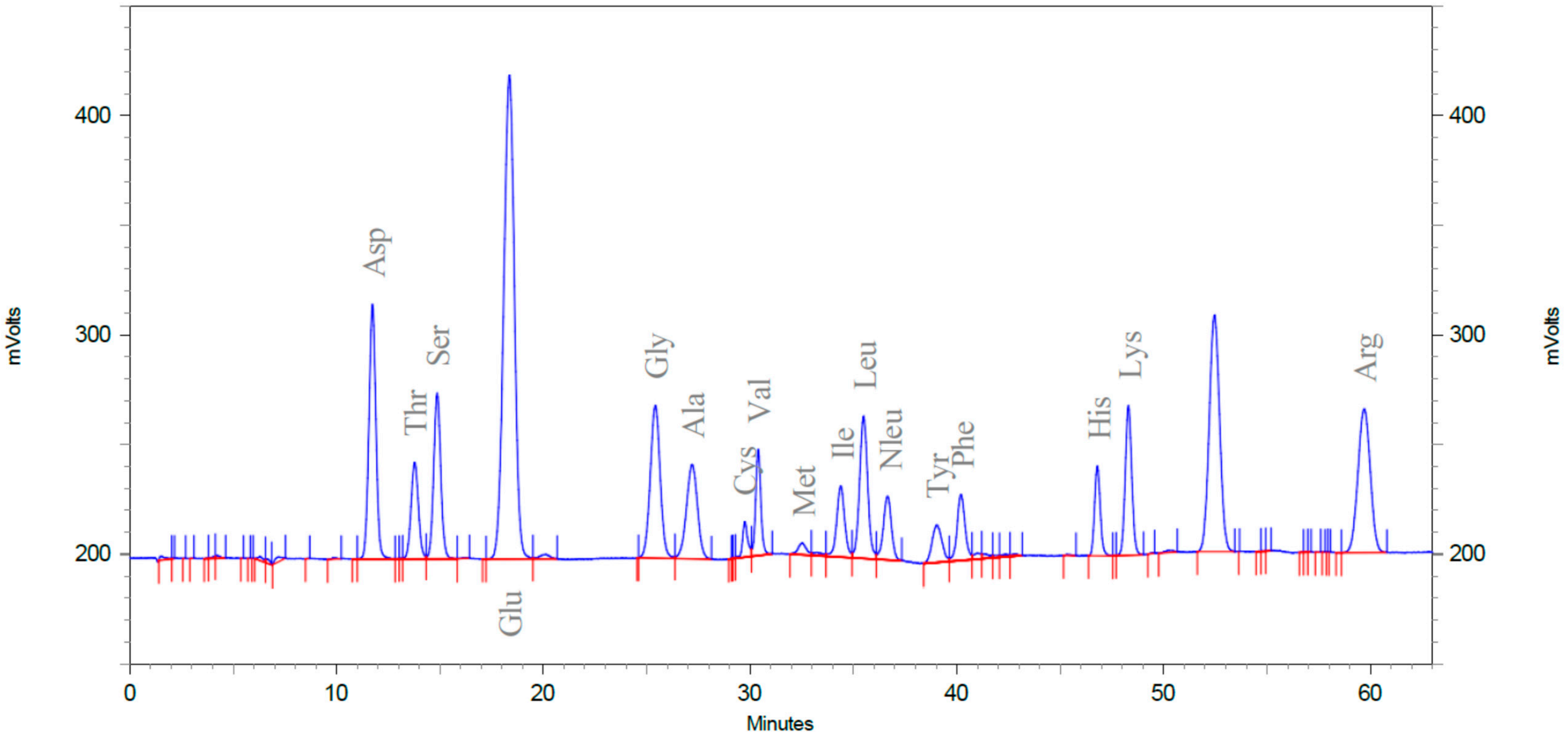

Figure S2: Chromatogram (amino acid analysis) of the albumin fraction of *Lupinus mutabilis* "Tarwi".

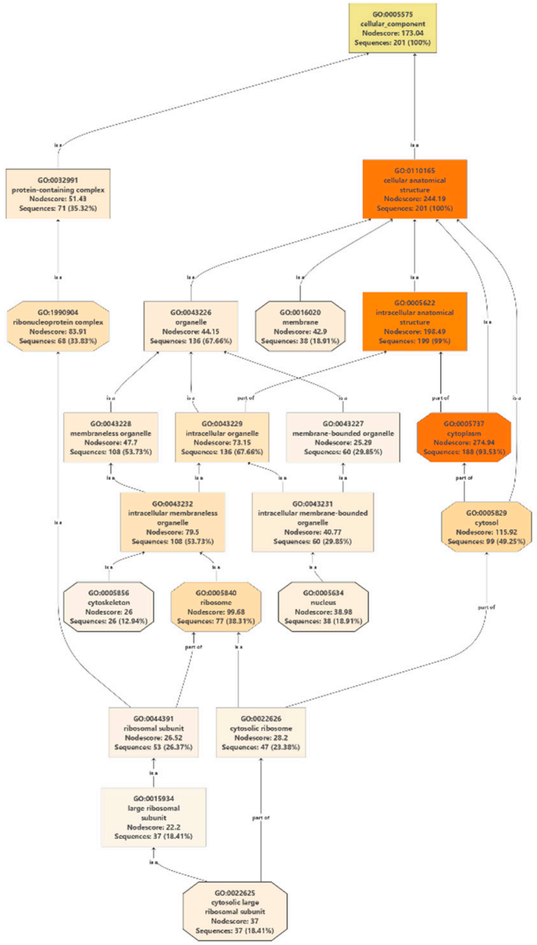

Figure S3: Complete functional distribution of detected proteins from the albumin fraction of *Lupinus mutabilis* in cellular component functional group using gene ontology (GO).

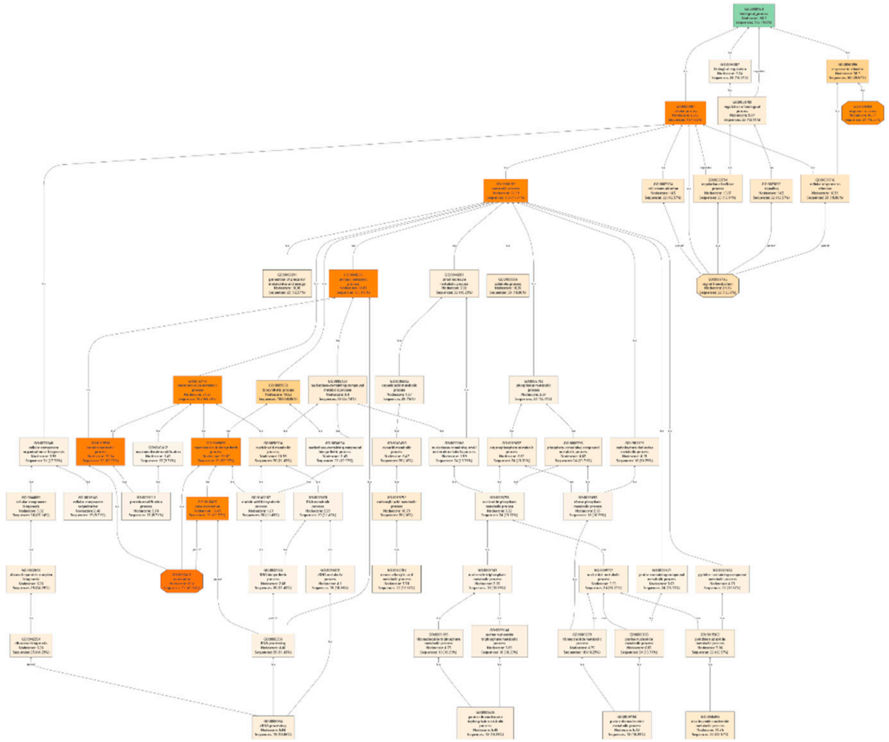

Figure S4: Complete functional distribution of detected proteins from the albumin fraction of *Lupinus mutabilis* in biological process functional group using gene ontology (GO).

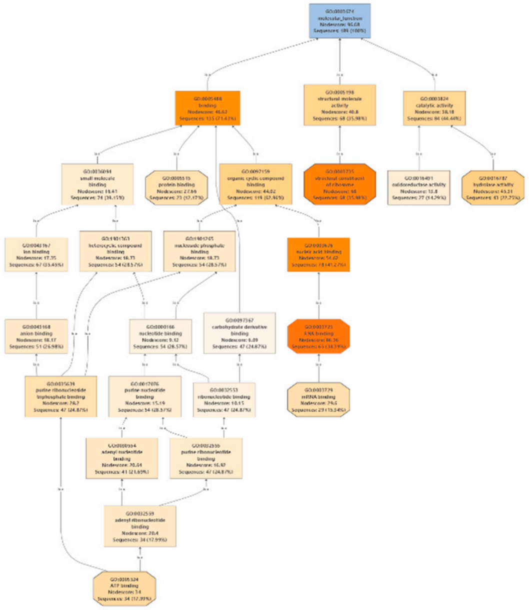

Figure S5: Complete functional distribution of detected proteins from the albumin fraction of *Lupinus mutabilis* in molecular function functional group using gene ontology (GO)

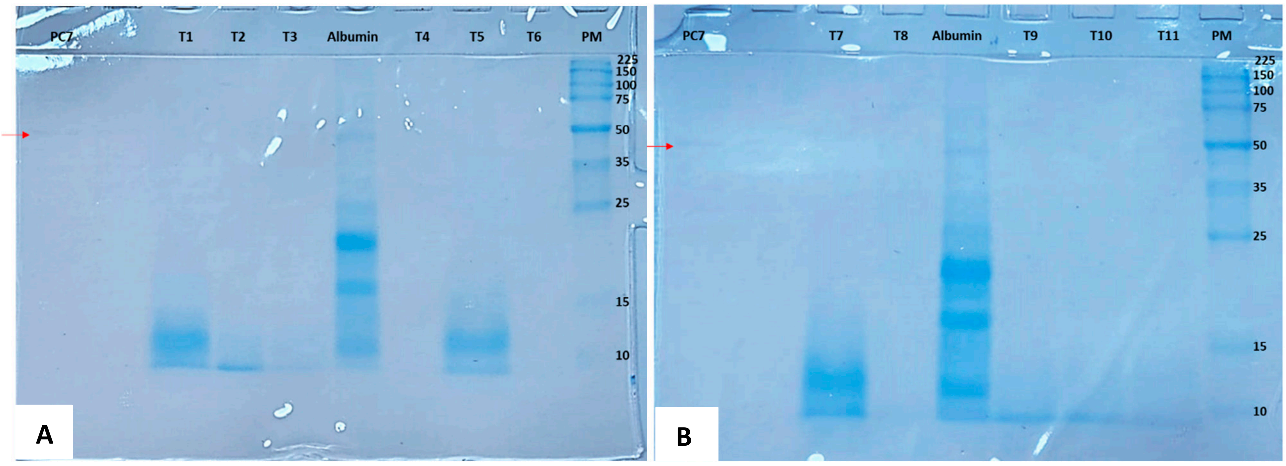

Figure S6. Electrophoretic profile (SDS-PAGE-Glycine) of the enzymatic hydrolysis optimization of the *Lupinus mutabilis* albumin fraction (AF) with PC7 protease. (A) Hydrolysis of treatments 1–6. (B) Hydrolysis of treatments 7–11. Albumin**:** Unhydrolysed AF fraction. MW**:** Molecular weight marker (Perfect Protein Marker, 10–225 kDa). The protein load per well was 10 µg.

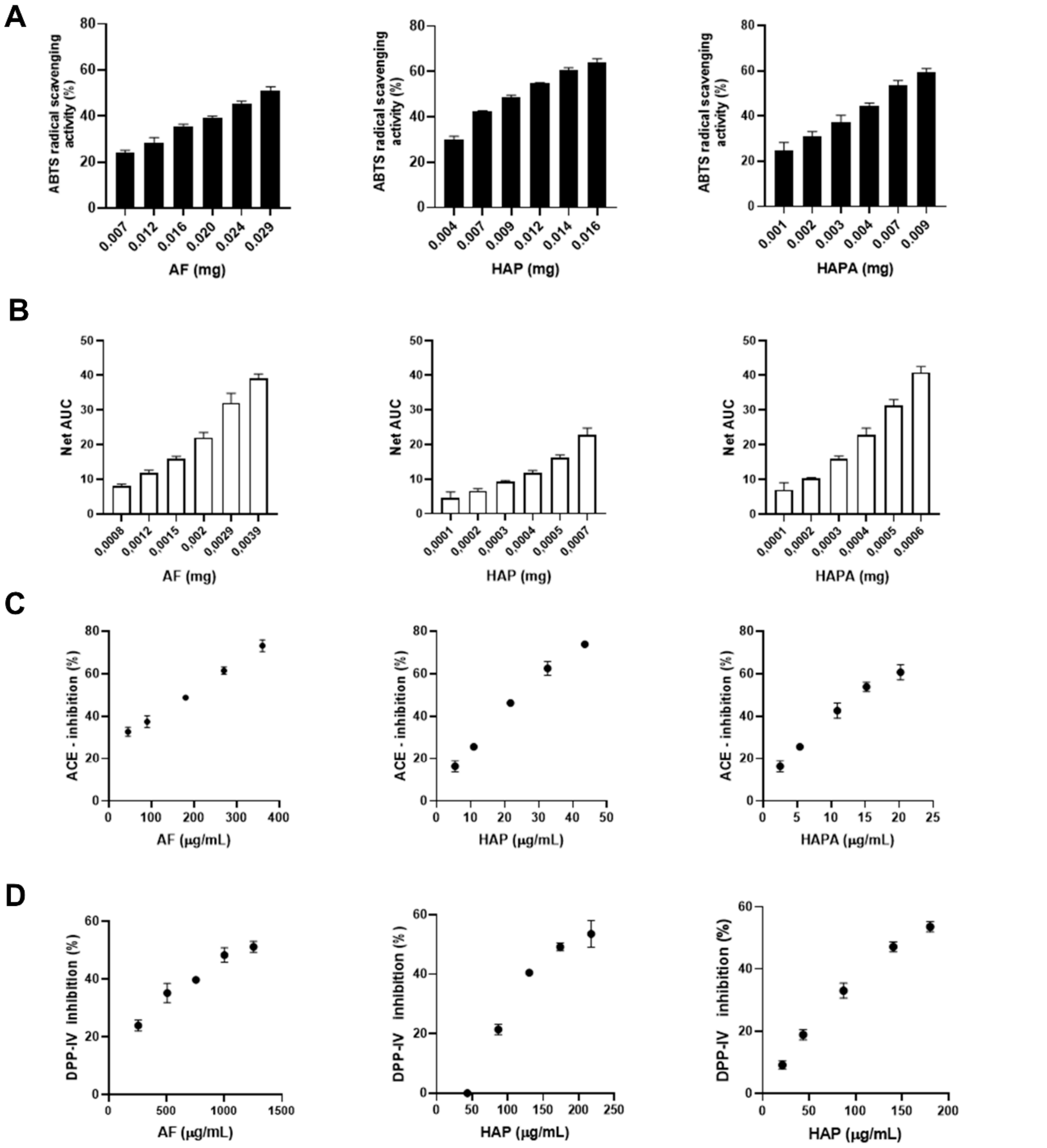

Figure S7: Evaluation of the bioactive properties of AF, HAP, and HAPA by the methods: A. ABTS, B. ORAC; C. ACE inhibition, and D. DPP-IV inhibition, at different sample concentrations. Where: AUC represents the area under the curve in the ORAC assay.

Table S1. Summary of key control parameters in the tangential ultrafiltration (TFF) process for concentrating albumin from *Lupinus mutabilis*.

Time (min)	Retentate pressure (PSI)	Feed Pressure (PSI)	Permeate flow (mL/min)	L/hr/m² (LMH)	TMP	NWP
5	20.5 ± 0.5	27.5 ± 0	18 ± 0	9.818 ± 0	24.00 ± 0.35	0.42 ± 0.0062
10	20.5 ± 0.5	27.5 ± 0	18 ± 0	10.364 ± 0	24.00 ± 0.35	0.42 ± 0.0062
15	20.5 ± 0.5	27.5 ± 0	18 ± 0	9.818 ± 0	24.00 ± 0.35	0.42 ± 0.0062
20	20.5 ± 0.5	27.8 ± 0.25	18 ± 0	9.818 ± 0	24.13 ± 0.53	0.42 ± 0.0092
25	21 ± 1.0	28.3 ± 0.75	19 ± 1	9.818 ± 0.77	24.63 ± 1.24	0.42 ± 0.0104
30	21.5 ± 1.5	28.8 ± 1.25	19 ± 1	9.818 ± 0.77	25.13 ± 1.94	0.42 ± 0.0013
35	22.5 ± 1.8	30.0 ± 1	19 ± 1	9.818 ± 0.77	26.13 ± 1.94	0.41 ± 0
40	22.5 ± 1.8	30.0 ± 1	19 ± 1	9.818 ± 0.77	26.13 ± 1.94	0.41± 0

TMP: Transmembrane Pressure; NWP: Normalized Water Permeability
